# The distribution of hrHPV genotypes among cervical cancer cases diagnosed across Ghana: a cross-sectional study

**DOI:** 10.1186/s12879-024-09166-7

**Published:** 2024-03-27

**Authors:** Patrick Kafui Akakpo, Emmanuel Gustav Imbeah, Kofi Ulzen-Appiah, Afua Darkwa-Abrahams, Ernest Adjei, Kwabena Amo-Antwi, Ernest Obeng Amo, Edmund Mounir Der, Edwin Kwame Wiredu

**Affiliations:** 1 Department of Pathology, School of Medical Science, University of Cape Coast / Cape Coast Teaching Hospital, Cape Coast, Ghana; 2 A.C.T Pathology consult, Pedu Estates, Pedu, Cape Coast, Ghana; 3 Department of Pathology, School of Medicine and Dentistry, University of Ghana/ Korle Bu Teaching Hospital, Korle Bu Accra, Ghana; 4 Department of Pathology, Komfo-Anokye Teaching Hospital, Kumasi, Ghana; 5 Department of Obstetrics and Gynecology, School of Medicine and Dentistry, Kwame Nkrumah University of Science and Technology, Kumasi, Ghana; 6 Pathologists Without Borders Ltd, Eduardo Mohdlana Street, Laterbiokorshie, Accra, Ghana; 7 Department of Pathology, School of Medical Sciences, University for Development Studies / Tamale Teaching Hospital, P.O Box TL, 1883 Tamale, Ghana

**Keywords:** Cervical cancer, HPV, Squamous cell carcinoma, Adenocarcinoma, Ghana

## Abstract

**Background:**

The burden of cervical cancer in Ghana is high due to a lack of a national screening and vaccination program. Geographical variations in high-risk Human Papilloma Virus incidence and type should be considered for vaccine improvement and screening in LMICs.

**Methods:**

A descriptive, multi-center cross-sectional study with purposive sampling of cases with cervical cancer diagnosed from January 2012 through to December 2018 was employed relying on archived Formalin Fixed Paraffin Embedded (FFPE) tissues from four (4) Teaching Hospitals. Cervical cancers were assessed for histopathological features following WHO guidelines. In addition, the novel Tumour Budding and Nest Size Grade (TBNS) for SCC, SILVA pattern of invasion for EAC and Tumour Infiltrating Lymphocytes (TILs) were assessed. High Risk HPV testing was performed using an isothermal, multiplex nucleic acid amplification method from ATILA biosystem (Mountain View California, USA). The FFPE blocks were tested for 15 hrHPV genotypes. Results were analyzed using SPSS v.26.0, with descriptive statistics and cross-tabulation and chi-square tests done with significance established at *p* < 0.05.

**Results:**

A total of 297 cases were identified for the study with ages ranging from 20 to 95 years. The peak age group for cervical cancer was 46 to 55 years. For those tested, hrHPV positivity rate was 85.4% [EAC (84.6%) and SCC (85.6%)]. The top five hrHPV serotypes for both histological cancers were 59 (40.0%), 35 (32.0%), 18 (30.0%), 16 (15.0%), and 33 (10.0%) respectively. Approximately, 58.2% of infections were multiple. Single hrHPV infections were mostly caused by hrHPV 59 (28.9%), and 16 (26.3%). TBNS grade for SCC, SILVA pattern of invasion for EAC and TILs did not show any statistically significant relationship with hrHPV.

**Conclusion:**

We affirm reported differences in hrHPV types associated with cervical cancer in Ghana with hrHPV types such as 59, 35, and 33 forming a significant proportion of hrHPV types associated with cervical cancer. This difference in hrHPV types should guide vaccine improvement and triaging of hrHPV positives. Though multiple infections are more common, some hrHPV types such as hrHPV 16 and 59 are responsible for most single infections associated with cervical cancer. Simple haematoxylin and eosin-based morphological assessments can improve the prognostication of patients with cervical cancer.

**Supplementary Information:**

The online version contains supplementary material available at 10.1186/s12879-024-09166-7.

## Background

Ghana has a population of 10.6 million women aged 15 years and above, who are at risk of developing cervical cancer. Cervical Cancer is the second most frequent cancer among women in Ghana, with an estimated 1699 women dying every year out of an estimated 2797 women diagnosed yearly with cervical cancer [1]. There is presently no national cervical cancer screening program, and screening is largely opportunistic. This contributes to the high burden of cervical cancer morbidity and mortality.

The causal link between high-risk human papilloma virus (hrHPV) genotypes and cervical cancer has been established. To date, up to 15 hrHPV types have been reported in the literature, as being associated with cervical precancer [2]. In this regard, there has been a shift from using PAP smear as a primary cervical precancer screening tool to hrHPV testing [2]. The World Health Organization’s (WHO) call for the elimination of cervical cancer is thus hinged on the vaccination of 90% of eligible girls by age 15 years against hrHPV, screening of 70% of eligible women at least twice in a lifetime (by 35 and 45 years) and preferably with hrHPV testing and treating 90% of screen-positive women and women with cervical cancer [3]. With this target achieved by 2030, the WHO projects that cervical cancer morbidity and mortality will be drastically reduced (median incidence reduced by 97% by 2120) [3].

The call to screen 90% of eligible women using hrHPV testing offers promise for many Low and Middle-Income Countries (LMICs) where resources for a traditional, PAP-based screening service are unavailable with a resulting high burden of cervical cancer. The options for hrHPV testing include full genotyping and partial genotyping depending on available resources [4]. High-risk HPV testing with partial genotyping has been reported to be equally effective in picking up cervical precancerous lesions and it is more cost effective when implemented in LMICs. Again, follow-up of screen positives may also be guided by the types of hrHPV detected at screening with more regular screening intervals recommended for infections with some hrHPV types such as hrHPV types 16 and 18, which are reported to be more associated with persistent infection and cancer [5, 6].

In line with the WHO’s call to vaccinate 90% of eligible girls by 15 years, the continuing success of such vaccination programs, when implemented in LMICs, depends on the use of improved vaccines that also target hrHPV types that are prevalent in these regions of the world. Presently, in sub-Saharan Africa, Rwanda has implemented a successful national vaccination program for adolescent girls 12 years and below, using existing vaccines that contain recognized common hrHPV types such as 16 and 18 [7]. While we await initial results on the effect of the vaccination program on cervical cancer mortality and morbidity, research into relevant hrHPV types, that cause cervical cancer in various regions of the world needs to be accelerated to inform vaccine improvement when outcome data become available.

Many published studies in Ghana, reported hrHPV types based on cervical samples collected during screening. These studies suggested variations in the types of hrHPV prevalent in our region [4–6, 8, 9]. They provided insight into hrHPV types prevalent in our region and such information may be useful for vaccine improvement. It is however crucial to also obtain such data from cervical cancers (CC) since the hrHPV types found in these lesions are indeed the candidate causative genotypes and thus require closer attention during vaccine improvement and triaging after hrHPV testing. Such studies are however lacking with only a few published so far and mostly from a single center [10–12]. Again, no study has yet used any of the automated hrHPV detection methods favoured for hrHPV testing in hrHPV-based CC screening in Ghana [13]. These methods offer the opportunity to test hrHPV as part of routine histopathological diagnostics in a prospective manner to aid in the prognostication of CC patients. We determined the prevalence and types of hrHPV in CCs using the AMPFIRE isothermal Polymerase Chain Reaction (PCR) detection system and correlated this with the histopathological characteristics of CCs.

## Methods

### Study design and site

This study is a descriptive, multi-center cross-sectional study with purposive sampling of cases from January 2012 through to December 2018. The study was conducted in the Department of Pathology, School of Medical Sciences, University of Cape Coast, Ghana and used archived Formalin Fixed Paraffin Embedded (FFPE) cervical cancer blocks retrieved from four Teaching Hospitals across Ghana, the Tamale Teaching Hospital (TTH), Cape Coast Teaching Hospital (CCTH), Komfo-Anokye Teaching Hospital (KATH), and Korle Bu Teaching Hospital (KBTH). These are located in the Southern, Middle and Northern belts of the country and are the major referral centers for cancer care.

### Inclusion and exclusion criteria

All cervical cancer cases with age stated and adequate tissue on available archived diagnostic FFPE blocks were included.

All cases with missing age and inadequate tissue on available FFPE blocks were excluded from the hrHPV testing. Again, all tissue with indeterminate results after DNA extraction and analysis were excluded.

### Sample collection and sample preparation

A search through the histopathology reports of the various teaching hospitals was initially carried out to identify all cases of CC diagnosed between January 2012 to December 2018. All old Haematoxylin and Eosin (H&E) stained slides of diagnosed CCs were initially screened by the PI (PKA) to confirm the diagnosis of CC. With inclusion met, patient records were de-identified.

FFPE tissue blocks of included cases were trimmed and an initial 10 μm section taken into Eppendorf tubes for hrHPV testing and genotyping using the AmpFire HPV High-Risk Genotyping kit from Atila biosystems Mountain View California. A final 4 μm deeper level was used to prepare new H&E slides for review to document the continued existence of CC and to document histopathological characteristics as part of the study. A new sterile microtome blade was used to section each case (FFPE block) to prevent cross-contamination. In addition, each section was picked with a disposable plastic forceps. Our method was a minor modification of an existing method that has been well documented in previous reports [7, 8].

### Histopathology slide review

To meet our objective of documenting histopathological characteristics of CC and correlating that with hrHPV status and type, all newly prepared H&E slides were reviewed by two (2) independent pathologists with graded experience (10 years and 4 years post specialist qualification). To start with, reporting protocols were agreed on, in addition to core data items (Appendix 1). Details of the Tumour Budding Nest Size (TBNS) grading system for Squamous Cell Carcinomas (SCC) [12, 13], new recommendations from the International Society of Gynaecological Pathologists on the reporting of endocervical adenocarcinoma and the SILVA pattern of invasion of endocervical adenocarcinomas were reviewed. To ensure that both pathologists understood the system and were able to use it, practice slides were provided with self-tests prior to the commencement of reporting [12, 14–16]. Additionally, slides were reported according to the old WHO 2014 guidelines (Appendix 1). In summary, the TBNS system relies on a combination of an assessment of tumour budding activity per ten (10) high power fields and smallest cell nest size within the tumour with size ranging from single cells to 15 cells. A summary table for the system is provided in appendix 1 [12, 13].

### HPV DNA purification, amplification and detection

Human papillomavirus-DNA isolation was performed with a commercially available kit (Atila BioSystems AmpFire HPV High-Risk Genotyping, V4.0 (Mountain View, CA)). AmpFire is a unique isothermal, multiplex nucleic acid amplification method where sequence-specific primers targeting each of the 15 hrHPV genotypes are used to amplify targeted sequences in the various HPV genotype regions. The amplified products interact with specific molecular beacon probes in real-time to generate fluorescence. The 15 high-risk HPV genotypes that can be detected individually with this system are (16, 18, 31, 33, 35, 39, 45, 51, 52, 53, 56, 58, 59, 66, and 68). The high-risk HPV specific primers and fluorescent probes are used to amplify regions of viral genomic DNA including E6/E7 regions and L1 proteins under isothermal conditions. The multiple proteins detected by the system helps to improve the sensitivity of the test which is also used for screening in our setting. The kit comprises the Reaction Mix (with buffer, enzymes, and dNTPs), Primer Mix (with primers and probes), Positive Control template, and Negative Control template [17]. Each assay includes a negative and positive control to ensure the quality of the assay performance and to rule out contamination. Only samples that passed both positive and negative controls were considered valid.

**Fig. 1:**
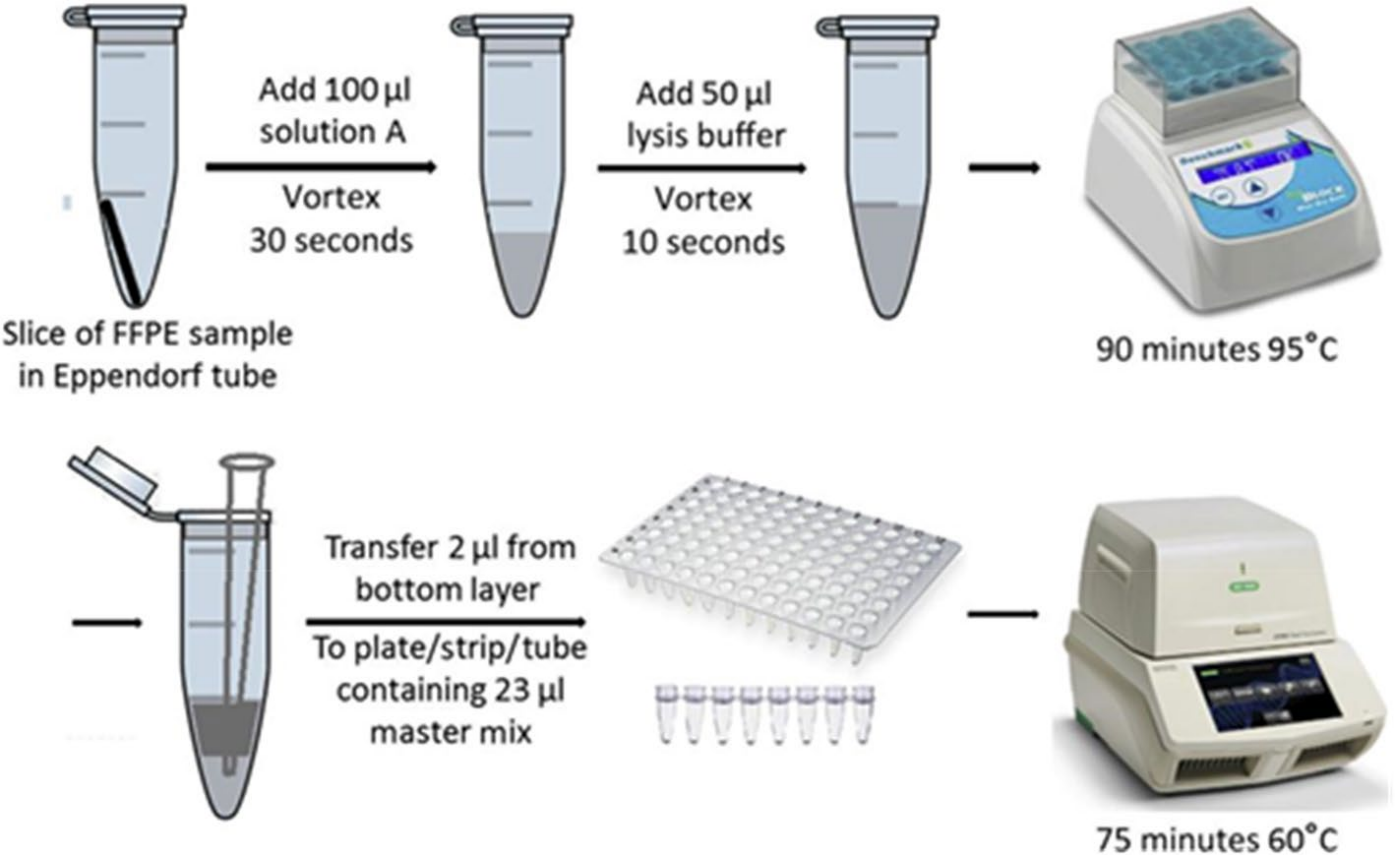
Diagram of AmpFire HPV assay procedures for formalin-fixed, paraffin-embedded (FFPE) samples. Briefly, slices of FFPE sample are treated with solution A and lysis buffer sequentially in a microcentrifuge tube. After 90-minute incubation at 95 °C, 2-μL solutions from the bottom layer in the micro-centrifuge tube are transferred and used directly in the AmpFire HPV assay [17]

Briefly, 10 μm slice of FFPE was taken and put in a 1.5 mL Eppendorf tube and 100 μL of Atila deparaffinizing solution (Solution A) added. This was vortexed to dissolve paraffin. 50 μL of lysis buffer was added and vortexed for 10 seconds to mix prior to spinning to obtain optimal sample lysis. The tube was incubated at 95 °C for 90 minutes and allowed to cool to room temperature before vortex and spin. Four (4) Master Mixes were prepared according to the manufacturer’s protocol and 20 μL of each of the Master Mixes were dispensed into the 96-well PCR plate. 2 μL of the specimen samples from the bottom layer were transferred to the corresponding wells. Again, 3 μL of distilled water was added to all the wells to bring the total volume to 25 μL. For the negative control reaction, 5 μL of the Negative Control template was transferred to the well. For the positive control reaction, 5 μL of the Positive Control Template was added to the well. All the wells were sealed with an optical compatible film and gently shaken to mix the reagents. The plate was then spun in a centrifuge to bring down all liquid to the bottom of the wells. The plate was subsequently put into the sample holder in the Atila Biosystem real-time PCR machine and the reaction run was started. The qualitative results generated in real time were recorded and analyzed. The processing method is presented in Fig. 1 below.

## Data analysis

Data analysis was carried out in SPSS Statistics software version 26 (IBM Corp., New York, USA) to obtain descriptive statistics. Categorical variables were examined for association by the Chi-square test. Continuous variables were tested for homogeneity of variances and normality before analysis. For categorical outcomes, proportions are presented as frequencies and percentages. For continuous variables, data is given as mean with corresponding standard deviation. Association between variables are done at a 95% confidence interval with significance established at *p <* 0.05*.*

## Results

A total of 297 cases were identified for the study with ages ranging from 20 to 95 years. The mean age for the study participants was 57.2 (SD ±14.6) years. The peak age group was 46 to 55 years, with just 1 (0.3%) woman below 25 years old. Most cases (49.8%) were from the Korle-Bu Teaching Hospital. Of all the cervical cancer cases, squamous cell carcinoma (SCC) represented 88.6% while adenocarcinoma was 11.4%. These are presented in Table 1.

**Table 1:** Demographic distribution of study samples

Characteristic	Variables	No. (%)
Age	Mean (SD)	57.23 (14.6)
Age Group (*N* = 297)		
	< 25	1 (0.3)
	26–35	18 (6.1)
	36–45	46 (15.5)
	46–55	82 (27.6)
	56–65	65 (21.9)
	66–75	48 (16.2)
	> 75	37 (12.5)
Facility (*N* = 297)		
	Tamale Teaching Hospital	37 (12.5)
	Komfo-Anokye Teaching Hospital	32 (10.8)
	Korle-Bu Teaching Hospital	148 (49.8)
	Cape Coast Teaching Hospital	80 (26.9)
Cancer type (*N* = 297)		
	SCC	263 (88.6)
	Adenocarcinoma	34 (11.4)

*SCC* Squamous Cell Carcinoma, *SD Standard Deviation

A comparison of age group and cancer type confirms the peak age group for both SCC and adenocarcinoma to be 46 to 55 years. However, the least age of diagnosis for SCC was less than 25 years while the least age of diagnosis for adenocarcinoma was between 36 years to 45 years. In the conventional grading system, most cervical cancers were classified as moderately differentiated followed by poorly differentiated and then lastly by well differentiated (170 vs 43 vs 34 cases respectively). This trend is very different when the TBNS system is employed depicting most cases as being poorly differentiated followed by moderately differentiated and then lastly by well differentiated (131 > 89 > 43 cases respectively). According to the SILVA pattern of invasion for adenocarcinoma classification, most cases are identified as Pattern C (26) as against Pattern B [7]. None of these findings compared by age group are however statistically significant. These are presented in Table 2.

**Table 2:** Comparison of age group against cancer type and grading systems

Age	Squamous cell Ca	Adenocarcinoma	Grade Conventional			Grade TBNS SCC/ SILVA Pattern For Adenocarcinoma					
						TBNS SCC			SILVA Pattern (Adenocarcinoma)		
			Well	Moderate	Poor	G1 - Well	G2 - Moderate	G3 – Poor	Pattern B	Pattern C	Cannot be determined
< 25	1 (0.4%)	0 (0%)	0 (0%)	1 (0.6%)	0 (0%)	1 (2.3%)	0 (0%)	0 (0%)	0 (0%)	0 (0%)	0 (0%)
26–35	18 (6.8%	0 (0%)	3 (8.8%)	12 (7.1%)	3 (5.1%)	3 (7%)	7 (7.9%)	8 (6.1%)	0 (0%)	(0%)	0 (0%)
36–45	39 (14.8%)	7 (20.6%)	6 (17.6%)	27 (15.9%)	6 (10.2%)	7 (16.3%)	13 (14.6%)	19 (14.5%)	0 (0%)	7 (26.9%)	0 (0%)
46–55	72 (27.4%)	10 (29.4%)	7 (20.6%)	47 (27.6%)	18 (30.5%)	12 (27.9%)	26 (29.2%)	34 (26.0%)	2 (28.6%)	7 (26.9%)	1 (100%)
56–65	56 (21.3)	9 (26.5%)	5 (14.7%)	35 (20.6%)	16 (27.1%)	6 (14.0%)	26 (29.2%)	24 (18.3%)	5 (71.4%)	4 (15.4%)	0 (0%)
66–75	42 (16.0%)	6 (17.6%)	8 (23.5%)	29 (17.1%)	5 (8.5%)	10 (23.3%)	12 (13.5%)	20 (15.3%)	0 (0%)	6 (23.1%)	0 (0%)
> 75	35 (13.3%)	2 (5.9%)	5 (14.7%)	19 (11.2%)	11 (18.6%)	4 (9.3%)	5 (5.6%)	26 (19.8%)	0 (0%)	2 (7.7%)	0 (0%)
Total	263 (100%)	34 (100%)	34 (100%)	170 (100%)	59 (100%)	43 (100%)	89 (100%)	131 (100%)	7 (100%)	26 (100%)	1 (100%)
*p-*value	0.557	0.557	0.619			0.060			0.106		

Analysis of the distribution of hrHPV indicates an overall 85.4% positivity as against 14.6% negative cases among cervical cancer cases (OR: 0.854, 95% CI: 0.800–0.899). Of all hrHPV-positive cases (*N* = 182) among CC, 160 (85.6%) were SCC while 22 (84.6%) were adenocarcinomas. Out of the 182 positive cases, 76 (41.8%) were single hrHPV infections while 106 (58.2%) showed multiple hrHPV infections (OR: 0.582, 95% CI: 0.507–0.655). Further analysis of the single hrHPV infections indicates hrHPV 59 (28.9%), and hrHPV 16 (26.3%) were the most predominant hrHPV types among the study population. Analysis of multiple hrHPV infections also indicates co-infection of hrHPV types 18, 35, 59 (26.4%) and hrHPV types 18, 35 (12.3%) as the predominant combinations, these are presented in Table 3.

**Table 3:** Frequency distribution of hrHPV characteristics among study samples

Characteristic	Variable	No. (%)
HPV status (*N* = 213)		
	Positive	182 (85.4)
	Negative	31 (14.6)
HPV Positives (*N* = 182)		
	SCC	160 (85.6)
	Adenocarcinoma	22 (84.6)
HPV Positivity (*N* = 182)		
	Single Infections	76 (41.8)
	Multiple Infections	106 (58.2)
Single HPV Infections (*N* = 76)		
(Top 6 Single infections)	HPV 16	20 (26.3)
	HPV 18	5 (6.6)
	HPV 33	6 (7.9)
	HPV 45	5 (6.6)
	HPV 59	22 (28.9)
	HPV 68	5 (6.6)
	Others with frequency < 5.5%	13 (17.1)
Multiple HPV Infections (*N* = 106)		
(Top 6 Multiple infections)	18,35	13 (12.3)
	18,35,59	28 (26.4)
	31,18,35,59	5 (4.7)
	35,59	3 (2.8)
	35,59,56,58	3 (2.8)
	45,66	4 (3.8)
	Others with frequency < 2.0%	50 (47.2)

The distribution of hrHPV positives among cervical cancer types are presented in Table 4. hrHPV 59 was the most predominant type recorded in SCC, accounting for 75 (40.1%) of all the 187 positive hrHPV cases with SCC. The other common hrHPV types in SCC were hrHPV 18, hrHPV 35, and hrHPV 16 recording 59 (31.6%), 59 (31.6%), and 29 (15.5%) respectively. Among the adenocarcinomas, the most predominant hrHPV type was HPV 59 with 11 (42.3%). The other common hrHPV types among adenocarcinoma included HPV 35, HPV 33, HPV 18, HPV 45, HPV 66, and HPV 68 comprising 8 (30.8%), 6 (23.1%), 5 (19.2%), 5 (19.2%), 5 (19.2%), and 5 (19.2%) respectively. There was a statistically significant correlation between HPV 33 positivity and cancer type (OR: 0.312, 95% CI: 0.110, 0.888. *p* = 0.035) with the odds favouring adenocarcinoma. All other variables were not statistically significant. Our study was however limited by the number of cases studied.

**Table 4:** Distribution of HPV Positives among the Cervical Cancer types in the study population

	Cancer Type		Total	
HPV Positive	SCC	Adenocarcinoma		*p-*value
HPV59	75 (40.1%)	11 (42.3%)	86 (40.4%)	0.495
HPV35	59 (31.6%)	8 (30.8%)	67 (31.5%)	0.566
HPV18	59 (31.6%)	5 (19.2%)	64 (30%)	0.145
HPV16	29 (15.5%)	3 (11.5%)	32 (15%)	0.426
HPV33	16 (8.6%)	6 (23.1%)	22 (10.3%)	**0.035**
HPV45	17 (9.1%)	5 (19.2%)	22 (10.3%)	0.110
HPV66	17 (9.1%)	5 (19.2%)	22 (10.3%)	0.110
HPV68	15 (8%)	5 (19.2%)	20 (9.4%)	0.078
HPV52	15 (8%)	1 (3.8%)	16 (7.5%)	0.392
HPV58	9 (4.8%)	0 (0%)	9 (4.2%)	0.302
HPV56	6 (3.2%)	2 (7.7%)	8 (3.8%)	0.253
HPV31	6 (3.2%)	1 (3.8%)	7 (3.3%)	0.604
HPV39	5 (2.7%)	0 (0%)	5 (2.3%)	0.518
HPV51	4 (2.1%)	0 (0%)	4 (1.9%)	0.592
HPV53	1 (0.5%)	1 (3.8%)	2 (0.9%)	0.230
Total (Within HPV)	187 (87.8%)	26 (12.2%)	213 (100.0%)	

A crosstabulation of hrHPV infections and grading parameters in EAC and SCC with TILS assessment are presented in Table 5. HPV 59 was the most predominant hrHPV type among EAC (42.3%). Majority of HPV 59 associated adenocarcinoma showed SILVA Pattern C invasion (47.6%). SILVA pattern C invasion is established to be associated with poor prognosis and late stage presentation and will be in line with reported late stage presentation of cervical cancer in Ghana. Similarly for SCC, HPV 59 was the most predominant HPV detected(40.1%). However, the majority of HPV 59 associated SCCs were moderately differentiated. Assessment of TILS among HPV 59 associated tumours showed intermediate TILs. Each of these associations were however not statistically significant. hrHPV 33 however showed a statistical significance correlation (*p* = 0.035) with tumour grade, accounting for 8.6% of HPV-associated SCC with majority (19.4%) being well differentiated (Grade 1).

**Table 5:** Histopathological characteristics of hrHPV Positives EAC and SCC

	Silva pattern of stromal invasion (for HPV A EAC only)					Tumour grading (Total score (TB + NS)) for SCC					Tumour TILS				
	Pattern B	Pattern C	Cannot be determined	Total	*p-*value	Well-differentiated (G1)	Moderately differentiated (G2)	Poorly differentiated (G3)	Total	*p-*value	Low (0–10%)	Intermediate (10–50%)	High (50–90%)	Total	*p-*value
HPV 16	1 (25%)	2 (9.5%)	0 (0%)	3 (11.5%)	*0.630*	7 (22.6%)	11 (18.3%)	11 (11.5%)	29 (15.5%)	*0.253*	23 (14.6%)	7 (16.3%)	2 (16.7%)	32 (15%)	*0.949*
HPV31	0 (0%)	1 (4.8%)	0 (0%)	1 (3.8%)	*0.884*	1 (3.2%)	3 (5%)	2 (2.1%)	6 (3.2%)	*0.603*	4 (2.5%)	3 (7%)	0 (0%)	7 (3.3%)	*0.282*
HPV39	0 (0%)	0 (0%)	0 (0%)	0 (0%)	*–*	0 (0%)	2 (3.3%)	3 (3.1%)	5 (2.7%)	*0.598*	4 (2.5%)	0 (0%)	1 (8.3%)	5 (2.3%)	*0.231*
HPV51	0 (0%)	0 (0%)	0 (0%)	0 (0%)	*–*	1 (3.2%)	2 (3.3%)	1 (1%)	4 (2.1%)	*0.567*	3 (1.9%)	0 (0%)	1 (8.3%)	4 (1.9%)	*0.171*
HPV18	0 (0%)	5 (23.8%)	0 (0%)	5 (19.2%)	*0.479*	7 (22.6%)	21 (35%)	31 (32.3%)	59 (31.6%)	*0.470*	45 (28.5%)	15 (34.9%)	4 (33.3%)	64 (30%)	*0.696*
HPV35	0 (0%)	7 (33.3%)	1 (100%)	8 (30.8%)	*0.129*	7 (22.6%)	21 (35%)	31 (32.3%)	59 (31.6%)	*0.470*	49 (31%)	14 (32.6%)	4 (33.3%)	67 (31.5%)	*0.971*
HPV59	0 (0%)	10 (47.6%)	1 (100%)	11 (42.3%)	*0.103*	15 (48.4%)	27 (45%)	33 (34.4%)	75 (40.1%)	*0.247*	66 (41.8%)	18 (41.9%)	2 (16.7%)	86 (40.4%)	*0.227*
HPV68	1 (25%)	4 (19%)	0 (0%)	5 (19.2%)	*0.850*	2 (6.5%)	3 (5%)	10 (10.4%)	15 (8%)	*0.451*	17 (10.8%)	3 (7%)	0 (0%)	20 (9.4%)	*0.389*
HPV33	1 (25%)	5 (23.8%)	0 (0%)	6 (23.1%)	*0.854*	6 (19.4%)	2 (3.3%)	8 (8.3%)	16 (8.6%)	***0.035***	15 (9.5%)	6 (14%)	1 (8.3%)	22 (10.3%)	*0.677*
HPV45	0 (0%)	5 (23.8%)	0 (0%)	5 (19.2%)	*0.479*	3 (9.7%)	4 (6.7%)	10 (10.4%)	17 (9.1%)	*0.725*	15 (9.5%)	5 (11.6%)	2 (16.7%)	22 (10.3%)	*0.698*
HPV66	0 (0%)	5 (23.8%)	0 (0%)	5 (19.2%)	*0.479*	4 (12.9%)	4 (6.7%)	9 (9.4%)	17 (9.1%)	*0.612*	16 (10.1%)	5 (11.6%)	1 (8.3%)	22 (10.3%)	*0.934*
HPV52	0 (0%)	1 (4.8%)	0 (0%)	1 (3.8%)	*0.884*	1 (3.2%)	4 (6.7%)	10 (10.4%)	15 (8%)	*0.394*	10 (6.3%)	4 (9.3%)	2 (16.7%)	16 (7.5%)	*0.375*
HPV53	0 (0%)	1 (4.8%)	0 (0%)	1 (3.8%)	*0.884*	0 (0%)	0 (0%)	1 (1%)	1 (0.5%)	*0.621*	1 (0.6%)	1 (2.3%)	0 (0%)	2 (0.9%)	*0.559*
HPV56	0 (0%)	2 (9.5%)	0 (0%)	2 (7.7%)	*0.773*	2 (6.5%)	2 (3.3%)	2 (2.1%)	6 (3.2%)	*0.486*	7 (4.4%)	1 (2.3%)	0 (0%)	8 (3.8%)	*0.634*
HPV58	0 (0%)	0 (0%)	0 (0%)	0 (0%)	*–*	4 (12.9%)	2 (3.3%)	3 (3.1%)	9 (4.8%)	*0.070*	8 (5.1%)	1 (2.3%)	0 (0%)	9 (4.2%)	*0.552*

## Discussion

A total of 297 histopathologically confirmed cervical cancer cases were retrieved from four teaching hospitals distributed as follows: Tamale Teaching Hospital (TTH); 37 (12.5%), Komfo-Anokye Teaching Hospital (KATH); 32 (10.8%), Korle-Bu Teaching Hospital (KBTH); 148 (49.8%) and Cape Coast Teaching Hospital (CCTH); 80 (26.9%) respectively. Out of this number, 263 (88.6%) were SCC, while 34 (11.4%) were adenocarcinomas. The current value of 11.4% being that for adenocarcinoma falls within the 7–29% [10–12] range reported in previous studies. The mean age of patients was 57.2 (SD ± 14.6) years. Histopathologically, there is little consensus on the grading of SCC and Adenocarcinomas of the cervix [12, 13, 18]. In relation to SCC, using a conventional grading system that relies on the presence of keratin, 65% of the cases were graded as moderately differentiated. However, when a more recently proposed system that relies on assessment and combined scoring of Tumour Budding and Nest Size (TBNS) [12, 13] was used to grade SCCs, 50% of the cases were graded as grade III (poorly differentiated) 34% as grade II (moderately differentiated) and 16% as Grade I (well differentiated). This finding better explains the poor prognosis of cervical cancers reported in Ghana and is in line with reports that a high TBNS grade is associated with worse outcomes. The TBNS system relies on the assessment of a combination of morphological parameters instead of just the presence of keratinization and thus better relates to prognosis compared to the traditional grading system [12, 13]. Its relationship with the aetiology of cervical cancer is however still under study. Improvements in the TBNS grading system may improve the usefulness of cancer grading in SCC, making it a robust adjunct to hrHPV presence and staging.

The SILVA pattern of invasion has been used as a surrogate for the depth of stromal invasion which also relates to stage and thus prognosis in hrHPV-associated adenocarcinoma. It relies on the presence of destructive stromal invasion and its extent combined with other factors such as lymphovascular space invasion [14, 18]. It allows for therapeutic surgical planning and prognostication to be carried out on punch biopsies and has been proven to relate to outcome [14], We applied the SILVA system to all adenocarcinomas irrespective of hrHPV status. All adenocarcinomas showed either SILVA pattern B (21%) or C (76%) invasion in line with the documented late-stage at presentation of cervical cancer patients in Ghana and the generally poor survival [19]. The emergence of immunotherapy has improved the prognosis of many cancers and holds promise for the management of cervical cancer. In line with this, many novel methods have been proposed for the evaluation of Tumour Infiltrating Lymphocytes (TILs) in cervical cancer so that patients could benefit from immunotherapy and improve their outcome [20, 21]. We carried out a simple assessment of TILs and graded the proportion of TILs as Low, Intermediate or High, with most tumours showing low TILs. A comparison of TBNS grading for SCC, SILVA pattern of invasion for adenocarcinomas and proportion of TILs for both cancer types to hrHPV positivity and type of hrHPV for the cases that had hrHPV testing, however, did not show any statistically significant relationship in our study. A more detailed study with a larger cohort of patients, comparing the morphological features of SCC and hrHPV status may also lay the foundation for a reporting system that relies on morphology and its relationship with hrHPV positivity as is now the case for cervical adenocarcinomas which are now reported based on hrHPV association [15, 16, 19, 20]. These morphological parameters if well-established will improve the management and outcome of patients diagnosed with CC, ultimately resulting in the achievement of the WHO 90: 70: 90 call in relation to the treatment of 90% of women who are screen positive or have cancer.

Two hundred and thirteen (213) CCs had adequate tissue and good molecular material and were tested for the presence of hrHPV. For those tested, the overall hrHPV positivity rate was 85.4%, in line with the rates for SCC (85.6%) and adenocarcinomas (84.6%). This is close to rates reported in some previous publications in Ghana [7, 8]. In their study, Awua et al. (2016) also reported identical rates of hrHPV positivity between SCC and adenocarcinoma [7]. Though others have quoted generally higher rates [8, 9, 12]. However, in both reports that quoted higher rates, the number of cases studied was lower. The almost identical hrHPV rates in adenocarcinomas are in line with other reports, notwithstanding that it has previously been reported that hrHPV rates were significantly lower in adenocarcinomas, our study did not find this and rather support an earlier finding by Awua et al. [7]. Overall, our relatively lower rate of hrHPV in CC may be due to the duration of storage of some of the FFPE blocks used, some of which dated back to 2012 and may not have been stored under optimal conditions. Out of the 15 hrHPV types detected, the top 10 in descending order were hrHPV 59 (40%), 35 (32%), 18 (30%), 16 (15%), 33 (10%), 45 (10%), 66 (10%), 68 (9%), 52 (8%), and 58 (4%). Though not in the same order, hrHPV 59, 18 and 16 were reported among the top 5 hrHPVs in a previous study [7]. In their study, hrHPV 59 was the second most common after hrHPV 18. However, hrHPV 45 and 31 were the other hrHPV types in the top five. Though the higher prevalence of hrHPV 59 and 35 have previously been reported, our study is the first to report it as the top two hrHPV types in CC. In line with our findings, many screening-based studies have reported a higher prevalence of hrHPV 35 and 59 [4–6, 10]. Obiri-Yeboah et al. reported an association between hrHPV35 and the presence of Squamous Intraepithelial Lesions (SIL) [5]. Within the context of cross-protection offered by belonging to the same phylogenetic tree/ family, vaccine improvement should be guided by the geographical variations in hrHPV types. Again, though these same hrHPV types have been reported to varying proportions in screening-based studies, the emphasis has been on traditional hrHPV types such as 16 and 18 and calls for the use of partial genotyping tests for hrHPV testing have been based on the use of existing hrHPV testing kits that detect hrHPV 16 and 18 as standalone types and combine all other hrHPV types as others. In this regard, the presence of some hrHPV types such as 59, and 35 may need to be determined in addition to the traditional 16 and 18 to help better triage patients who are hrHPV positive for follow-up. This is of paramount importance when hrHPV-based screening relies on partial hrHPV genotyping, an approach that has been proposed for LMICs. It will allow for better risk stratification of hrHPV positives for follow-up screening.

As has been reported in many publications from Ghana, multiple infections with hrHPV were more common in our study than single infections (58.2% vs 48.1%). The majority of hrHPV types were significantly more likely to be detected with other hrHPV types. This was the case for all the hrHPV types except hrHPV types 16 and 59 which accounted for most of the single infections, the rest being accounted for by the other hrHPV types except hrHPV types 31, 53 and 56 which never occurred as single infections. In this regard, hrHPV 59 had a higher likelihood of being present as a single infection even when compared to hrHPV 16 (12% vs 11%). This finding suggests that the determination of the presence of hrHPV type 59 may be as crucial as the determination of hrHPV types 16 and 18 and that consideration should be given to kits that identify hrHPV 59 as a standalone type during partial genotyping, an approach that is currently being promoted in LMICs due to cost [4, 6]. Though hrHPV type 16, was not as common as hrHPV types 59 and 18 in our cohort, it tended to exist alone when it was determined in CCs thus suggesting that it may be more virulent and able to cause persistent (latent) disease resulting in CC and thus once detected should result in patient stratification into a higher risk category. This will support arguments for partial genotyping that rely on the determination of hrHPV type 16 as a standalone hrHPV type to aid risk stratification and follow-up. From our top five hrHPV types, 33 accounted for 3.3% of single infections compared to 35 (1.1%) and 18 (2.7%). These were, however, not statistically significant.

## Limitations

Our study is limited by preanalytical conditions and sample processing during the preparation of the Formalin Fixed Paraffin Embedded (FFPE) tissue, sample size and, use of a single genotyping assay and the length of storage of the FFPE tissue blocks used. Though the assay selected for this study is one that has been routinely used, validated and comparatively analyzed in previous studies of smears, our study is limited by our sole use of that assay [6, 7, 10].

## Conclusion

We affirm reported differences in hrHPV types associated with cervical cancer in Ghana with hrHPV types such as 59, 35, and 33 forming a significant proportion of hrHPV types associated with cervical cancer. This difference in hrHPV types should guide vaccine improvement and triaging of hrHPV positives. Though multiple infections are more common, some hrHPV types such as hrHPV 16 and 59 are responsible for most single infections associated with cervical cancer. Simple haematoxylin and eosin-based morphological assessments can improve the prognostication of patients with cervical cancer. These deductions are made acknowledging various limitations in the study. Further research related to novel morphology-based hrHPV related typing and grading of SCC and adenocarcinomas of the cervix is crucial, especially in LMICs where routine hrHPV testing is not available.

### Supplementary Information

**Supplementary Material 1.**

**Supplementary Material 2.**

**Supplementary Material 3.**

## Data Availability

All materials used in the study are available and attached as appendices. Reporting template used in the study is provided as Appendix 1. Grading system employed in the study is presented as Appendix 2. Protocol for Ampfire HPV high risk genotyping as used in this study is provided in Appendix 3.

## Abbreviations

SCC: Squamous cell carcinoma
hrHPV: High risk human papilloma virus
EAC: Endocervical adenocarcinoma
TBNS: Tumour budding and nest size
CC: Cervical cancer
SIL: Squamous intraepithelial lesion
