# Supplementary material for: The distribution of hrHPV genotypes among cervical cancer cases diagnosed across Ghana: a cross-sectional study
Source: BMC Infect Dis. 2024 Mar 27;24:356. doi: 10.1186/s12879-024-09166-7 (PMC10967043; doi:10.1186/s12879-024-09166-7)
Supplement: Supplementary file 1 — Supplementary Material 1. [file 12879_2024_9166_MOESM1_ESM.docx]

APPENDIX 1
