# Supplementary material for: The distribution of hrHPV genotypes among cervical cancer cases diagnosed across Ghana: a cross-sectional study
Source: BMC Infect Dis. 2024 Mar 27;24:356. doi: 10.1186/s12879-024-09166-7 (PMC10967043; doi:10.1186/s12879-024-09166-7)
Supplement: Supplementary file 2 — Supplementary Material 2. [file 12879_2024_9166_MOESM2_ESM.docx]

**APPENDIX 2**

TBNS grading system^12,13^.

| **Grading proposal for SCC of the uterine cervix** | **Score** |
| --- | --- |
| **Tumour budding activity/10 HPF** No budding < 15 budding foci ≥ 15 budding foci **Smallest cell nest size within the tumour core** > 15 cells 5–15 cells 2–4 cells Single cell invasion | 1 2 3  1 2 3 4 |
| **Tumour grading** Well-differentiated (G1) Moderately-differentiated (G2) Poorly differentiated (G3) | **Total score** 2–3 4–5 6–7 |
