# Supplementary material for: The distribution of hrHPV genotypes among cervical cancer cases diagnosed across Ghana: a cross-sectional study
Source: BMC Infect Dis. 2024 Mar 27;24:356. doi: 10.1186/s12879-024-09166-7 (PMC10967043; doi:10.1186/s12879-024-09166-7)
Supplement: Supplementary file 3 — Supplementary Material 3. [file 12879_2024_9166_MOESM3_ESM.docx]

**APPENDIX 3:**

**Ampfire HPV High Risk Genotyping Protocol on FFPE Samples**
